# Exploring differential evolution for inverse QSAR analysis

**DOI:** 10.12688/f1000research.12228.2

**Published:** 2017-09-06

**Authors:** Tomoyuki Miyao, Kimito Funatsu, Jürgen Bajorath

**Affiliations:** 1 Department of Chemical System Engineering, School of Engineering, The University of Tokyo, Tokyo, 113-8656, Japan; 2 Department of Life Science Informatics, B-IT, LIMES Program Unit Chemical Biology and Medicinal Chemistry, Rheinische Friedrich-Wilhelms-Universität, Bonn, D-53113, Germany

**Keywords:** Chemical space, active compounds, differential evolution, support vector regression, virtual screening, inverse QSAR

## Abstract

Inverse quantitative structure-activity relationship (QSAR) modeling encompasses the generation of compound structures from values of descriptors corresponding to high activity predicted with a given QSAR model. Structure generation proceeds from descriptor coordinates optimized for activity prediction. Herein, we concentrate on the first phase of the inverse QSAR process and introduce a new methodology for coordinate optimization, termed differential evolution (DE), that originated from computer science and engineering. Using simulation and compound activity data, we demonstrate that DE in combination with support vector regression (SVR) yields effective and robust predictions of optimized coordinates satisfying model constraints and requirements. For different compound activity classes, optimized coordinates are obtained that exclusively map to regions of high activity in feature space, represent novel positions for structure generation, and are chemically meaningful.

## Introduction

Inverse quantitative structure-activity relationship (QSAR) analysis aims to identify values of descriptors used to generate a QSAR model that corresponds to high activity, and build structures of active compounds from these values ^1– 4^. The inverse QSAR process is challenging since numerical signatures of activity, if they can be determined, must be re-translated into viable chemical structures and active compounds, a task falling into the area of *de novo* compound design ^5– 7^. A predominant approach to inverse QSAR is the use of multiple linear regression (MLR) models to construct chemical graphs that correspond to an MLR equation ^1– 4^. Given this equation, a desired y (activity) value constrains relationships between descriptor settings. These constraints make it possible to derive vertex degree or edge sequences, from which chemical graphs might be constructed. For instance, specialized descriptors have been introduced for inverse QSAR on the basis of MLR equations and algorithms for constructing chemical graphs from these descriptors ^8– 11^. So far only few inverse QSAR studies have employed methods other than MLR. For example, it was attempted to construct chemical graphs from the centroid of activity of a set of compounds in Hilbert space defined by a kernel function ^12^. In this case, a pre-image approximation algorithm was used to obtain coordinates in descriptor space and construct chemical graphs from these descriptor coordinates. Alternatively, inverse QSAR was divided into a two-stage process by separating the derivation of preferred descriptor values for a desired activity from the chemical graph construction phase ^13– 15^. Descriptor information corresponding to a given y value was represented via probability density functions, and regression analysis was performed using Gaussian mixture models in combination with cluster-wise MLR ^14^. Subsequently, chemical graphs satisfying a set of descriptor values, or ranges of descriptor values, were generated by assembling ring systems and atom fragments with monotonically changing descriptors ^14^. Following this approach, descriptor values must increase when adding an atom, ring system, or other structural fragment to a growing chemical graph. Applying Gaussian mixture models and cluster-wise MLR makes it possible to focus on the applicability domain ^14, 15^ of the underlying models.

The two-stage inverse QSAR process is conceptually based on an important premise adopted from conventional (forward) QSAR, i.e., the higher a predicted activity value is, the more desirable a chemical structure becomes. In two-stage inverse QSAR, this conjecture challenges the descriptor value generation phase because value combinations are ultimately desired that correspond to higher predicted activity than exhibited by any currently available training or test compound. In other words, descriptor settings should be optimized for predicted activity. For this purpose, the use of Gaussian mixture models and cluster-wise MLR left considerable room for improvement, due to its multi-parametric nature and tendency of overfitting if training data were organized into large number of clusters ^14^. Recently, autoencoder modeling was proposed as an approach for two-stage inverse QSAR ^16^. Continuous latent space, corresponding to a descriptor space, is constructed on the basis of encoding a line notation of a molecule by recurrent neural networks (RNNs). Following this methodology, optimized coordinates in latent space can be directly translated into another line notation by the decoder consisting of RNNs. As such, the approach does not depend on chosen descriptors and has the potential to automatically address two-stage inverse QSAR in a single step. However, the generation of new valid line notations (SMILES strings) for chemical structures corresponding to optimized coordinates was difficult in a case study designing organic light-emitting diodes ^16^.

In this work, the descriptor optimization challenge of two-stage inverse QSAR has been specifically addressed. We emphasize that the chemical graph construction phase of inverse QSAR is not subject of this work and beyond its scope. Rather, our focal point has been the development of a new methodology for optimizing descriptor settings with respect to higher than observed compound activity, as a prerequisite for candidate structure generation. Therefore, an evolutionary approach is introduced to identify descriptor coordinates that correspond to the highest predicted activity within the applicability domain of a given QSAR model. The methodology and results of proof-of-concept studies are presented in the following.

## Methods

### Methodological concept

**Figure 1.**
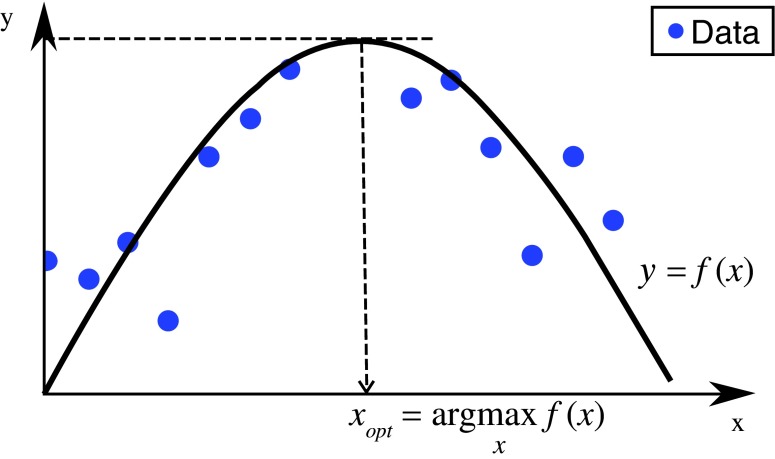
Optimization concept. A regression function f( **x**) fits training data to determine new coordinates in descriptor space. Optimized coordinates based on f( **x**) fall inside the training data range but yield a higher y value than any other data point.

Inverse QSAR depends on the derivation of descriptor coordinates for a given model and data set. The goal of the methodology presented herein is finding desirable coordinates in a pre-defined descriptor space ( **x** space) on the basis of a regression function f( **x**) representing a QSAR model. Confining the search to the applicability domain (AD) of the model translates this task into a constrained optimization problem (COP). The concept of the optimization is illustrated in Figure 1. Newly derived coordinates should be more desirable with respect to pre-defined evaluation criteria than any other data point used to construct the regression model. Accordingly, COP is formulated as follows:

Minimize f( **x**)

$$\begin{array}{l} \text{s}.\text{t}.\;{\text{g}}_{\text{j}}(\text{x})\leq 0,\text{j}=1,\ldots ,\text{q}\\ \;{\text{h}}_{\text{j}}(\text{x})=0,\text{j}=\text{q}+1,\ldots ,\text{m}\\ \;\text{x}=({\text{x}}_{1},\ldots ,{\text{x}}_{\text{d}}),{\text{l}}_{\text{i}}\leq {\text{x}}_{\text{i}}\leq {\text{u}}_{\text{i}},\text{i}=1,\ldots ,\text{d} \end{array}$$

where **x** ∈ R ^d^, f: R ^d^ → R is the function to be optimized, g _j_: R ^d^ → R is the j-th inequality function, and h _j_: R ^d^ → R is the j-th equality function. The i-th component of **x**: x _i_ falls into the range [l _i_, u _i_].

For the purpose of our analysis, the following assignments are made:

**x:** descriptors;

–f( **x**): QSAR model;

–g _1_( **x**): AD model;

g _k_( **x**) (k = 2, …, j), h( **x**): constraints for descriptors;

l _i_, u _i_: lower and upper bounds of i-th descriptor.

Constraints are applied to descriptors to ensure meaningful value ranges. For example, if the ‘number of heavy atoms’ (x _p_) and ‘number of hydrogen bond acceptors’ (x _q_) are selected descriptors, a value of five for the former and six for the latter would be impossible for any given data point (compound). Therefore, in order to prevent such settings, an inequality constraint is required and applied: x _q_ – x _p_ ≤ 0.

### ε Differential evolution

**Figure 2.**
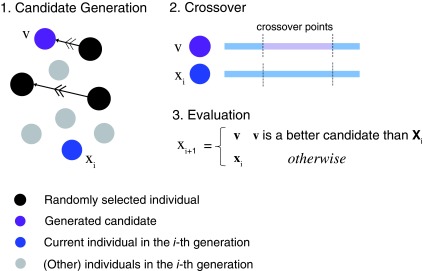
Evolutionary algorithm. The steps involved in evolutionary optimization are outlined. First, a candidate **v** is obtained from three randomly selected individuals by a differential operation. Second, a crossover operation is applied to an individual **x**_i_ and the candidate. Third, the evaluation step involves ε level comparison of **v** and **x**_i_ and results in the next individual.

For addressing COP, the differential evolution (DE) algorithm originally introduced by Stone and Price ^17^ is investigated herein, which has so far not been considered in inverse QSAR. However, given the conceptual simplicity and computational efficiency of DE, the algorithm has been successfully applied to solve optimization problems in other areas of science and engineering, for example, in scheduling of flow shops ^18^. In addition, for deriving a COP solution efficiently, ε differential evolution (εDE) was introduced by Takahama *et al.* as an extension of DE ^19^, illustrated in Figure 2. A candidate vector **v** for next generation (also called mutant vector) is derived on the basis of three randomly selected vectors:

$$\text{v}={\text{x}}_{\text{r}1}+\text{F}({\text{x}}_{\text{r}2}-{\text{x}}_{\text{r}3}),$$

where **x**_r1_, **x**_r2_, **x**_r3_ are different vectors from the current generation and F represents a scale parameter for the difference vector. If a variable adopts only discrete values, its value in **v** can be rounded to an integer. If the i-th component of **v**: v _i_ falls outside the range [l _i_, u _i_], **v** is updated as follows: ^4^

$${\text{v}}_{i}=\{\begin{array}{l} \text{min}\;\{{\text{u}}_{i},{\text{l}}_{i}+({\text{l}}_{i}-{\text{v}}_{i})\},\;\text{if}\;{\text{v}}_{i}<{\text{l}}_{i}\\ \text{max}\;\{{\text{l}}_{i},{\text{u}}_{i}-({\text{v}}_{i}-{\text{u}}_{i})\},\;\text{if}\;{\text{u}}_{i}<{\text{v}}_{i} \end{array}.$$

An exponential crossover operation with probability-based crossover points is applied to **v** (the probability is called CR). Either **x**_i_ or **v** is selected as **x**_i+1_, the individual for the next generation, following ε level comparison of the corresponding vectors.

### ε Level comparison

For prioritizing candidates, given constraints and the optimized function are taken into account. The constraint violation Φ( **x**) is defined as follows:

$$\Phi (x)=\sum_{j=1}^{q}\text{max}\;\{0,g_{j}\left(x\right){\}}^{p}+{\sum_{j=q+1}^{m}\left|h_{j}(x)\right|}^{p},$$

where Φ( **x**) represents the degree of violation, with p set to one. The ε level comparison determines the order between two sets of pair (f( **x**), Φ( **x**)):

$$(f_{1},\Phi_{1}){<}_{\varepsilon }(f_{2},\Phi_{2})\Leftrightarrow \{\begin{array}{l} f_{1}<f_{2},\;\text{if}\;\Phi_{1},\Phi_{2}\leq \varepsilon (t)\\ f_{1}<f_{2},\;\text{if}\;\Phi_{1}=\Phi_{2}\\ \varphi_{1}<\varphi_{2},\;otherwise \end{array},$$

where t represents a generation in DE. As a decreasing function of t, ε determines the tolerance of constraint violation and ε(t) is determined as follows: ^17^

$$\epsilon (t)=\{\begin{array}{ll} \Phi (x_{\theta }) & t=0\\ \Phi (x_{\theta }){\left(1-\frac{t}{T_{c}}\right)}^{cp} & 0<t<T_{c}\\ 0 & T_{c}\leq t \end{array}\;,$$

where **x**_θ_ is the top θ-th individual, T _c_ determines the generation in which ε(t) becomes zero, and cp the convergence speed. During the optimization, Φ( **x**) gradually outweighs f( **x**). In the initial stages of the optimization ε(t) settings enable the selection of diverse candidates but convergence of the algorithm is determined by Φ( **x**) becoming zero. Accordingly, T _c_ was set to one herein.

### Regression and applicability domain models

For εDE optimization, any regression function can be employed. In this study, support vector regression (SVR) ^20^ with ν parameter was applied and the AD was defined by one-class support vector machine (OCSVM) ^21^ classification with ν parameter. This parameter ranges from zero to one and defines the upper bound of the fraction of margin error and lower bound of the fraction of support vectors. AD consists of regions where the output of OCSVM is greater than or equal to zero. For both SVR and OCSVM, the radial basis function (RBF) kernel: k( **x**_*i*_, **x**_*j*_) = (−γ|| **x**_*i*_ − **x**_*j*_|| ^2^) was used. A hyper parameter set {C, ν, γ} for ν-SVR was determined by cross validation of training data on the basis of Q ^2^. For OCSVM model construction, γ was set to maximize the variance of the Gram matrix consisting of the kernel function of the training data ^22^ and ν was set to 0.01.

### Simulation data

Data points on a (x _1_, x _2_) plane were randomly generated for x _1_: [-2 3], x _2_: [-1, 4] to yield 50 training and 20 test instances. The corresponding y values were calculated using Mishra’s bird function ( https://mpra.ub.uni-muenchen.de/2718/) adding a Gaussian error with a mean of zero and variance of one, defined as:

$$\text{f}({\text{x}}_{1},{\text{x}}_{2})=\text{sin}({\text{x}}_{1})\text{exp}\{{\left(1-\text{cos}({\text{x}}_{2})\right)}^{2}\}+\text{cos}({\text{x}}_{2})\text{exp}\{{\left(1-\text{sin}({\text{x}}_{1})\right)}^{2}\}+{\left({\text{x}}_{1}-{\text{x}}_{2}\right)}^{2}.$$

**Figure 3.**
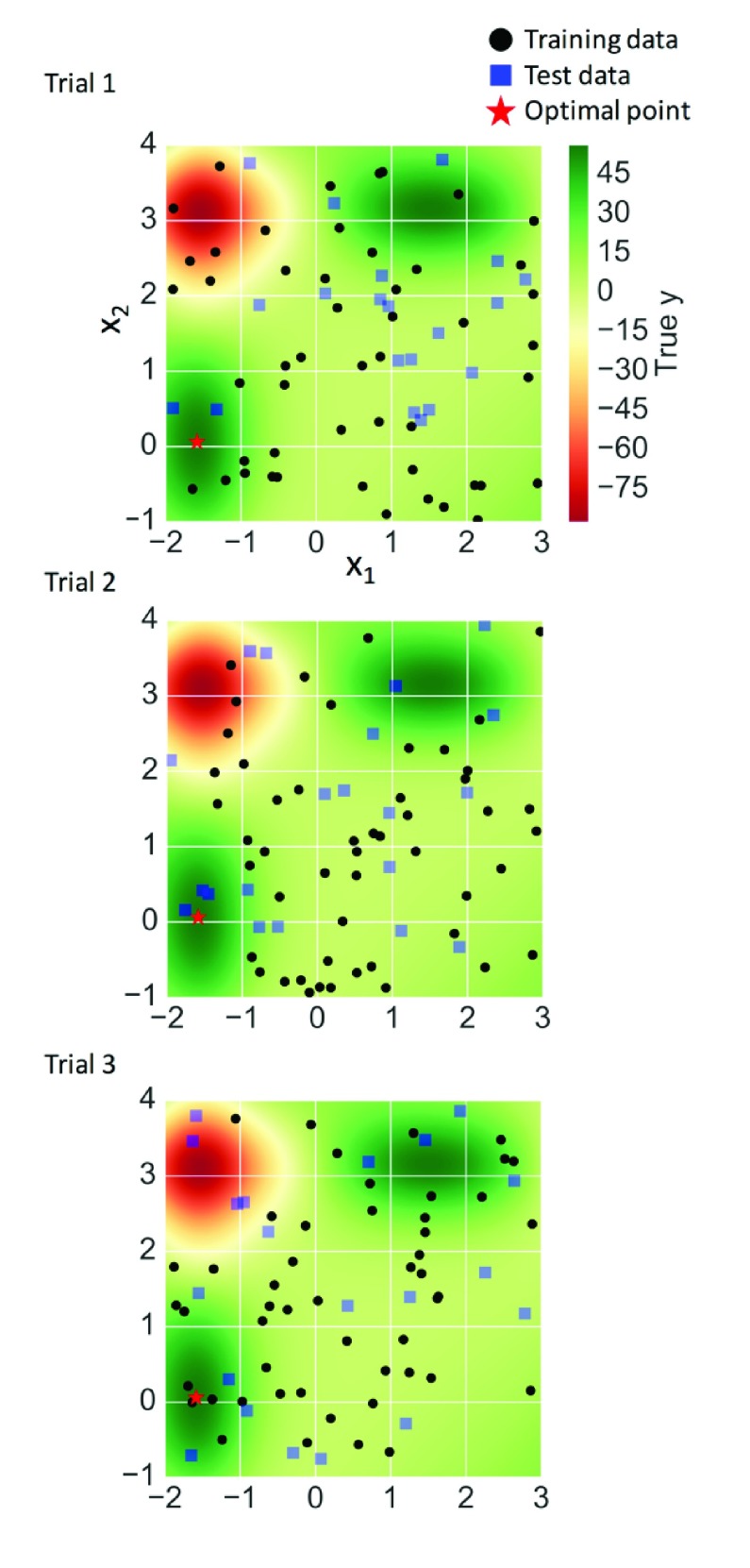
Simulation data sets. In three independent trials, simulation data sets were generated. For each trial, training (black dots) and test (blue squares) data are shown with true y values produced by the bird function f(x _1_, x _2_).

Three independent trials were carried out with different random number generators. Training and test data sets were plotted on the output domain of the bird function with color-coded *true y* values ( Figure 3).

### Compound data sets

From ChEMBL ^23^ (version 22), compound data sets were selected using the following criteria: Maximal assay confidence score of ‘9’, interaction relationship type ‘D’(direct), activity standard unit ‘nM’, activity standard type ‘K _i_’, and activity standard relation ‘=’. When multiple K _i_ values were available for a compound, their geometric mean was calculated to yield its final potency value, provided all measurements fell into the same order of magnitude (otherwise, the compound was discarded). In-house implementation of substructure filters for pan assay interference compounds (PAINS) ^24^ and other reactive molecules were applied to eliminate compounds with potential chemical liabilities. Filtering was not critical for modeling, but active compounds with sound chemical structures were desired. From qualifying data sets, nine activity classes were randomly selected, as summarized on Table 1.

**Table 1.** Compound data sets. Nine compound activity classes were taken from ChEMBL (version 22). For each activity class, the target ID (TID), number of compounds (CPDs) for which descriptor values were obtained, and number of descriptors following variable selection are reported.

TID	Activity class	# CPDs	# Descriptors
11	Thrombin inhibitors	1022	26
15	Carbonic anhydrase II inhibitors	2387	26
51	Serotonin 1a (5-HT1a) receptor ligands	1939	26
100	Norepinephrine transporter ligands	1179	26
107	Serotonin 2a (5-HT2a) receptor ligands	1570	26
194	Coagulation factor X inhibitors	1586	25
10193	Carbonic anhydrase I inhibitors	2380	26
12209	Carbonic anhydrase XII inhibitors	1750	26
12968	Orexin receptor 2 ligands	1041	29

For each compound, 44 descriptors were initially calculated using RDKit ( http://www.rdkit.org). These descriptors included constitutional descriptors (e.g., MW, number of rings, number of rotatable bonds), topological descriptors (e.g., Chi and Kier indices ^25^) and partial charge descriptors based on chemical graph’s topology (i.e., maximum of Gasteiger/Marsali partial charges ^26^). The set of 44 descriptors and the nine compound data sets used herein are made available in an open access deposition on the ZENODO platform ^27^. From correlated pairs of descriptors exceeding a correlation coefficient of 0.9, only one was chosen. For each activity class, the final number of descriptors (variables) is reported in Table 1. Compounds from each class were randomly divided into equally sized training and test data sets.

### Virtual screening

To test the ability of virtual screening (VS) to identify new active compounds from optimized coordinates, ChEMBL (version 22) was used as a screening source. From 1,414,176 unique compounds passing the substructure filters, training molecules used for modeling of each activity class were removed. All remaining ChEMBL compounds provided a large screening source for VS. For screening compounds, descriptors were calculated as described above and the compounds falling inside the AD of each class-specific model were preselected. Active compounds from each activity class not used for training represented true-positive test instances, regardless of their potency values. The calculation of descriptors for more than 1.4 million screening compounds was computationally demanding and exceeded the requirements for coordinate optimization.

For ChEMBL screening compounds including test instances, two VS ranking were generated. First, Euclidean distances to optimized coordinates were calculated. In this case, compound potency was not considered for ranking. Second, pK _i_ values were predicted for all screening compounds using the class-specific SVR models. The latter calculations were carried out to determine if true positives were highly ranked on the basis of activity predictions. The area under the receiver operating characteristic curves (AUC) was calculated as an evaluation criterion.

## Analysis protocol

Two proof-of-concept studies were carried out, one using simulation data, the other compounds and their activities. For simulation data, AD and regression models were constructed with the training data from each trial. Training data range scaling within the interval [-1,1] was applied prior to model building. For the SVR models, preferred parameter settings were determined using 10-fold cross validation. Coordinate optimization was carried using individual training data points. Optimized coordinates were evaluated on the basis of true y and maximal training data y values.

The same protocol for coordinate optimization was applied to each compound activity class. Furthermore, for hyper-parameter optimization of SVR, five-fold cross validation was carried out. For εDE, predicted pK _i_ values falling into the AD of each model were used to ensure that optimized coordinates were proximal to compound coordinates, as assessed by distance calculations. Furthermore, optimized coordinates were projected on principal component analysis (PCA) maps of the **x** space formed by the first and second PC. A matched molecular pair (MMP)-based ^28^ analog search for compounds proximal to the optimized coordinates was also carried out to investigate if analogs exhibited lower (higher) potency when they were distant from (close to) the optimized coordinates. Based on the distance to the optimized coordinates, the top 30 compounds from both training and test data sets were selected. MMPs qualified for this analysis if participating compounds displayed a potency difference of at least one order of magnitude.

The following εDE parameter settings were applied: Number of iterations, 1,000 and 10,000 for simulation and compounds data sets, respectively; F, 0.5; T _c_,1; p, 1; CR, 0.9 for all data sets. An initial population was obtained using 50 vectors of training instances for simulation data and 511 to 1,193 vectors for training compounds, depending on the size of the compound data sets.

Finally, the ability of distance- and SVR-based VS to predict new active compounds was analyzed. Although *de novo* structure generation was beyond the scope of our investigation, VS might be considered as an alternative way to identify novel active compounds, which was thus examined in the context of our study.

## Implementation

All SVR models and ADs were constructed with Scikit-learn ^29^ 0.18.1 using Python. εDE was implemented in C++. Descriptors were calculated using RDKit interfaced with Python.

All selected compound entries were standardized by removal of ions and solvent molecules and structure regularization, according to the OEChem toolkit (v1.7.7; OpenEye Scientific Software, Inc. Santa Fe, NM, USA).

## Results and Discussion

### Differential evolution for inverse QSAR

Optimization of descriptor coordinates for preferred values of a given model is a central aspect of the two-stage inverse QSAR process, for which currently only approximate solutions exist. Therefore, a more accurate methodology for coordinate optimization is highly desirable, as investigated herein. The evaluation of εDE as an optimization method for this critical task was inspired by previous results obtained for other types of optimization problems where this approach displayed better performance than alternative evolutionary methods, such as genetic algorithms or particle swarm optimization ^30– 32^. Moreover, ε-based lexicological comparison of individual feature vectors makes this algorithm straightforward to apply to problems where several constraints must be balanced, as is the case in inverse QSAR. Studies on simulation and compound data sets were designed to evaluate whether εDE was capable of effectively optimizing coordinates on the basis of a regression function.

### Investigating simulation data

For initial proof-of-concept, εDE-based search for optimized coordinates was carried out using simulation data generated as described above.

For the three simulation data sets, SVR models were built yielding optimized parameters {γ, ν, C} of {4, 0.25, 2}, {2, 0.25, 1}, {1, 0.125, 16}, respectively. For all OCSVM models, γ was 1. As reported in Table 2, these SVR models accounted for the output of the bird function in a statistically meaningful way.

**Table 2.** Derivation of the support vector regression model for simulation data sets. For each trial, model performance was assessed on the basis of R ^2^ and root mean square error (RMSE) values for training and test data sets.

Trial	Training		Test	
	R ^2^	RMSE	R ^2^	RMSE
1	1.00	1.10	0.87	5.17
2	0.96	3.22	0.70	12.73
3	0.97	3.17	0.84	11.99

**Figure 4.**
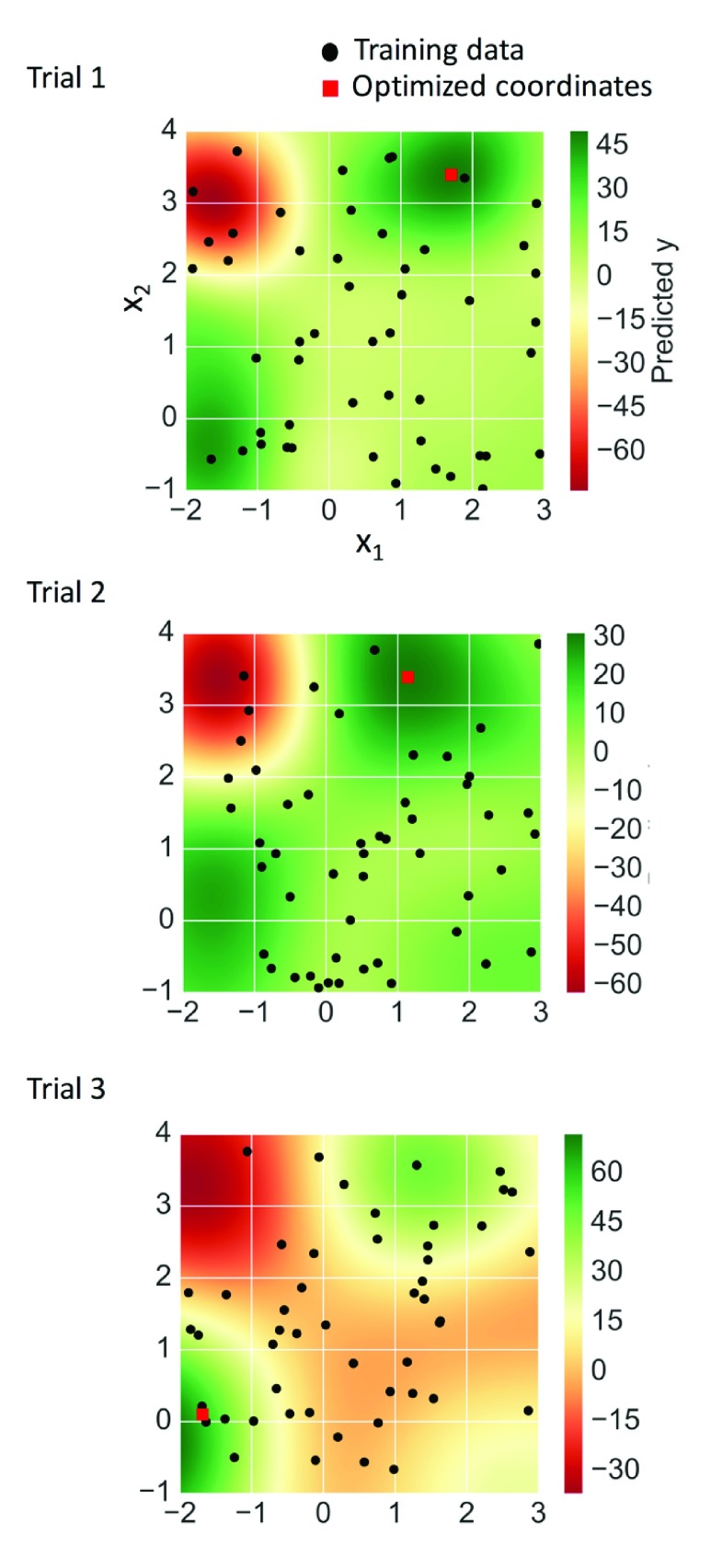
Optimized coordinates. For each of three independent trials, optimized coordinates (red squares) and training data (black dots) were mapped on the SVR prediction surface.

Standard deviations in the test data sets for trials 1, 2, and 3 were 14.55, 23.21, and 30.42, respectively. Figure 4 shows the different prediction surfaces of the SVR models for the three trial sets. The surfaces of set one and two were overall similar, whereas the surface of set three differed from the others. In each case, however, individual vectors converged at a single point ( Table 3) and optimized coordinates were located in regions of highest predicted y values ( Figure 4). In set one, for which the SVR model overall best accounted for the bird function, a training data point was found adjacent to the optimized coordinates, which slightly exceeded the largest predicted y value ( Table 3).

**Table 3.** Prediction of y values. For each simulation data trial, y values predicted by the SVR model are reported. For training data, the largest measured y value is given in parentheses. “Domain” is defined by x _1_ and x _2_ with a resolution of 0.005. AD refers to the applicability domain of the OCSVM model. For optimized coordinates, the result of the bird function is given in parentheses as the true y value. (i.e., the y value without error).

Trial	Training ( *measured y*)	Domain	AD	Optimized coordinates ( *true y*)
1	48.92 (50.02)	50.19	50.19	50.19 (49.54)
2	26.22 (29.46)	31.14	31.14	31.14 (48.09)
3	58.87 (55.18)	71.94	59.40	59.41 (55.15)

For set two, no training data were mapped to local maxima of the bird function, which resulted in a difficult regression scenario. The maximal measured y value in the training data was 29.46 and for optimized coordinates (1.51, 3.16), the predicted y value was 31.14, also slightly exceeding the largest measured y value. However, the predicted value was much smaller than the corresponding true y value of 48.09 ( Table 3), due to the inherent regression limitation.

For set three, the maximal true y value within the domain was 56.18 at (-1.59, 0.06). In this case, several training data points were mapped to regions of y values into which optimized coordinates fell ( Figure 4), leading to an extrapolative over-prediction of the corresponding y value of 71.94. However, this over-prediction was correctly balanced when the AD of the model was considered instead, leading to a value of 59.40 and a predicted y value for the optimized coordinates of 59.41 ( Table 3).

Despite typical regression limitations highlighted by findings for set two and three, the results obtained for simulation data indicated the potential of εDE for predicting optimized coordinates. Importantly, all solutions converged to single vectors representing novel points in simulation data space with large predicted y values falling inside the AD. Taken together, these results indicated the principal potential of εDE for coordinate optimization on the basis of SVR modeling.

### Coordinate optimization for compound data sets

Next εDE optimization was applied to different compound activity classes. In each case, SVR models were derived, optimized coordinates determined, and activity values predicted.

For each compound class, optimized coordinates yielded larger predicted pK _i_ values than any training or test set compound ( Table 4), consistent with the methodological strategy. Optimized coordinates fell inside the AD of each model and were proximal to several active compounds. When setting the ν parameter value to 0.1, hence restricting compound numbers within the AD, optimized coordinates remained unchanged compared to the ν parameter setting of 0.01. Nearest neighbors of optimized coordinates were mostly predicted to be highly active ( Table 4), indicating the presence of smooth prediction surfaces in the vicinity of optimized coordinates.

**Table 4.** Optimized coordinates and nearest neighbors. For optimized coordinates, the predicted pK _i_ value and the output of the OCSVM model for the applicability domain (AD) are reported. For training and test instances, the predicted pK _i_ value and scaled distance from optimized coordinates are given for the nearest neighbor (NN).

TID	Optimized coordinates		NN in training data		NN in test data	
	Predicted pK _i_	AD	Distance	Predicted pK _i_	Distance	Predicted pK _i_
11	11.49	0.26	0.61	9.50	0.52	10.02
15	12.20	0.06	0.46	10.21	0.45	10.32
51	10.25	0.62	0.39	9.24	0.40	9.09
100	9.50	0.22	0.32	8.63	0.46	7.97
107	11.43	0.32	0.71	9.78	0.71	9.84
194	13.03	0.17	0.82	10.92	0.67	11.90
10193	9.92	0.23	0.33	8.86	0.47	7.56
12209	10.48	0.35	0.72	8.99	0.78	8.69
12968	10.12	0.21	1.06	9.40	1.07	9.39

**Figure 5.**
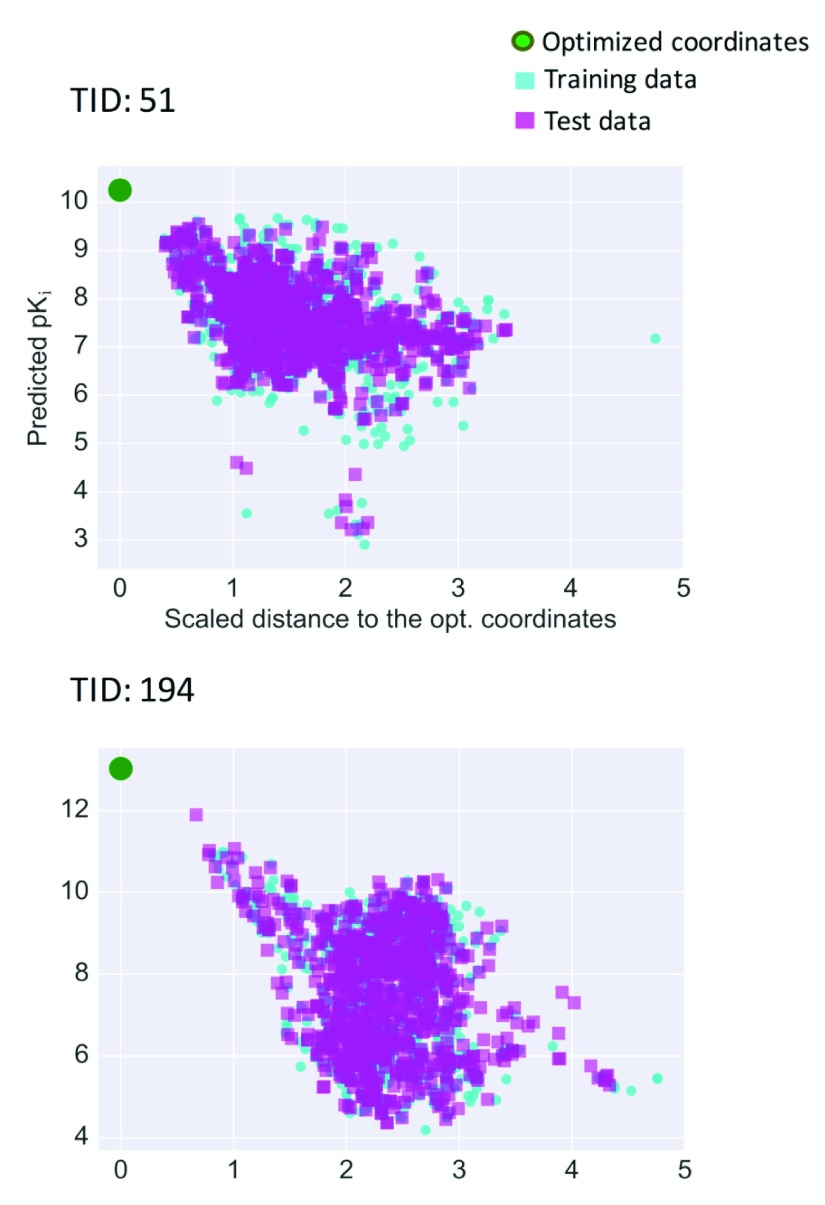
Activity prediction. For two exemplary activity classes, predicted pK _i_ values are related to the scaled distance of the corresponding compounds to the optimized coordinates. Training data (cyan squares), test data (magenta squares), and optimized coordinates (green circle) are shown.

Prediction surfaces were further characterized graphically by systematically comparing predicted pK _i_ values of compounds and calculated distances to optimized coordinates. Figure 5 shows the results for two exemplary activity classes, and Figure S1 shows the results for all classes. For set 51 (5-HT1a receptor ligands) in Figure 5, many highly active compounds were located proximal to the optimized coordinates, indicating that these coordinates fell into a well-populated region of activity-relevant chemical space. For set 194 (factor X inhibitors), training and test compounds tended to exhibit higher predicted pK _i_ with decreasing distance to the optimized coordinates, hence delineating regions of activity progression, which are relevant for compound optimization and exploitation of optimized coordinates.

**Figure 6.**
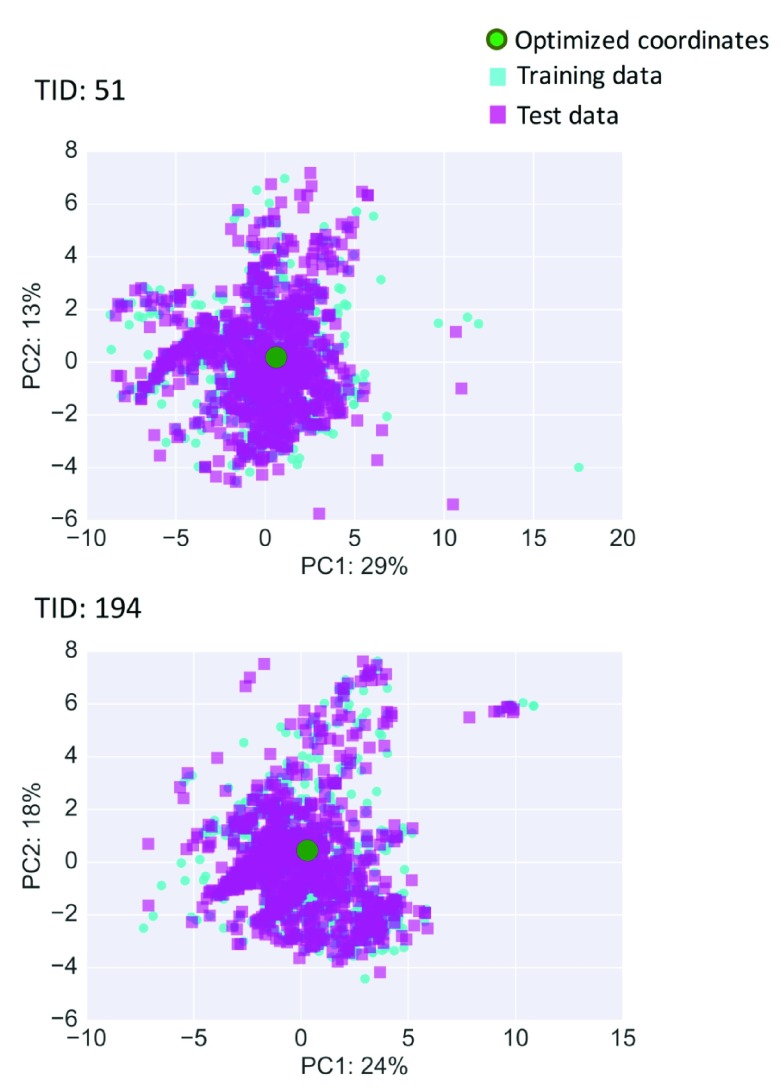
Projection of optimized coordinates. For the two activity classes from Figure 5, training data (cyan squares), test data (magenta squares), and optimized coordinates (green circle) were projected on a principal component (PC) plot derived from training data. For PC1 and PC2, contributions to data set variance are reported in %.

**Figure 7.**
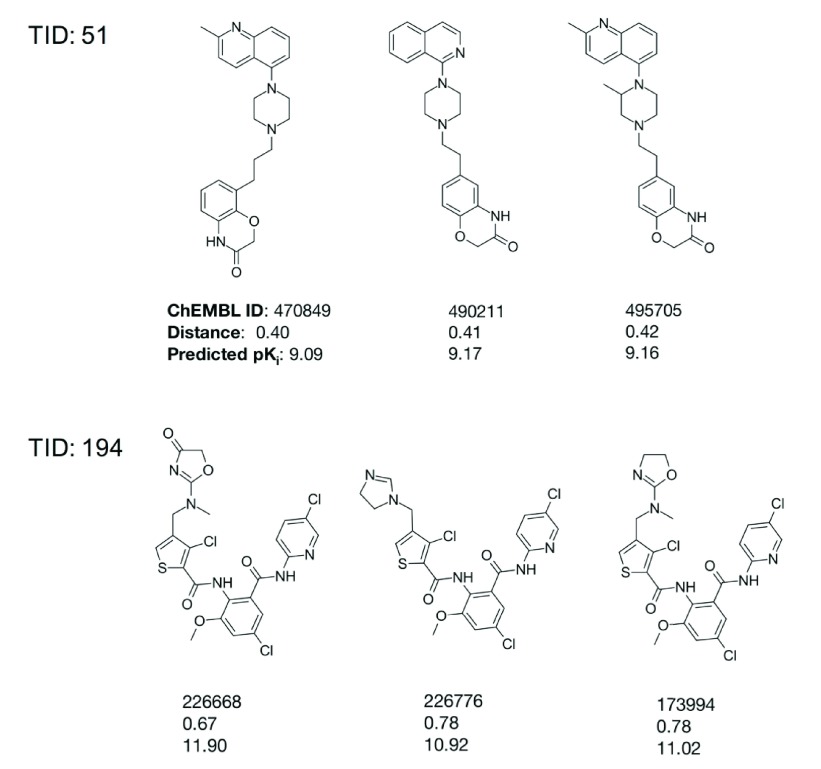
Nearest neighbors of optimized coordinates. For the two activity classes in Figure 5 and Figure 6, structures of the three nearest neighbors of optimized coordinates are shown and their ChEMBL IDs, scaled distances to the optimized coordinates and predicted pK _i_ values, are reported.

Data set compounds and optimized coordinates were also projected onto PCA plots of descriptor space ( Figure 6, Figure S2). These projections revealed that optimized coordinates were central to activity class regions in feature space. Furthermore, Figure 7 shows structures of the three nearest neighbors of the optimized coordinates for sets 51 and 194. In both cases, these compounds were structural analogs. Hence, similarity in feature space corresponded to close structural relationships. Consequently, this would also apply to structure generation from optimized coordinates, which would result in additional analog(s), consistent with the principles of QSAR and inverse QSAR.

A search was carried out for analogs of the top 30 compounds based on the distance to the optimized coordinates and the consistency ratio was calculated for each target ( Table 5). The number of qualifying MMPs ranged from 14 to 464. The consistency ratios were greater than 0.5 for eight of nine activity classes, implying that structures approaching optimized coordinates may have increasing potency.

**Table 5.** MMP-based analog search of the top 30 compounds. The consistency ratio and number of MMPs for the top 30 compounds based on the distance to the optimized coordinates are reported.

TID	Consistency ratio	# MMPs
11	0.62	253
15	0.71	68
51	0.88	108
100	0.64	14
107	0.33	93
194	0.86	464
10193	0.68	130
12209	0.75	60
12968	0.84	86

The εDE protocol can also be modified to generate a family of solutions. One possibility is splitting a data set into subsets of structurally related compounds, followed by the εDE optimization for finding the optimized coordinates on a per subset basis. Furthermore, since εDE is a methodology for solving a COP, it is also possible to incorporate multiple constraints as solubility and toxicity during the optimization process as long as these constraints are formulated as inequality equations using the same descriptors as for the objective function.

### Virtual screening

**Figure 8.**
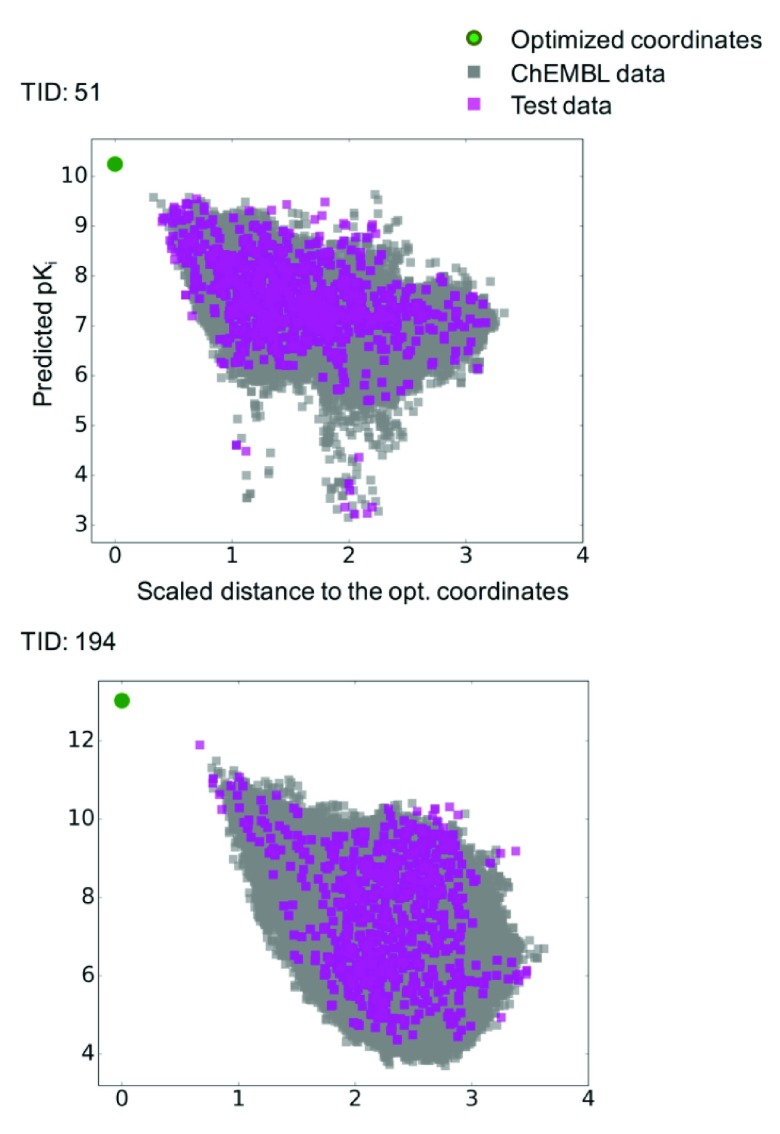
Activity prediction for ChEMBL compounds. For the two activity classes from Figure 5, predicted pK _i_ values are plotted against the scaled distance of corresponding compounds to the optimized coordinates. ChEMBL compounds (gray squares) and test compounds according to Figure 5 (magenta squares) falling inside the applicability domain are shown. Optimized coordinates are displayed as a green circle.

ChEMBL compounds were screened relative to optimized coordinates from the nine activity classes and Euclidian distances were determined. Furthermore, pK _i_ values were predicted for screening compounds falling into the AD of each class-specific SVR model. The AD was simply defined as the region where the OCSVM output was greater than or equal to zero. Since the nu value of the OCSVM models was set to 0.01, most of the training and test compounds were classified inside the AD. For example, for data set 51, 97% of the training and 96% of the test compounds fell inside the AD. The VS calculations ultimately led to alternative distance- and potency-based compound rankings. Table 6 reports screening compound statistics and VS results. For each activity class, screening compounds contained large numbers (497–1152) of true positive test instances. Distance-based VS yielded AUC values of at least 0.6 for five of nine activity classes, with a maximum of 0.76. For the four remaining classes, essentially random predictions were observed. Potency-based VS produced AUC values of greater than 0.6 for seven of nine classes, including values above 0.7 for three classes and a maximum of 0.77. Thus, potency-based predictions led to slightly better compound rankings than distance-based VS relative to optimized coordinates, but prediction accuracy was overall moderate at best. Moreover, the true positive ratio among the 30 top-ranked compounds was generally very low for both distance- and potency-based VS. Figure 8 shows exemplary potency prediction landscapes including optimized coordinates as a reference. Figure S3 shows these representations for all activity classes. Highly potent compounds proximal to optimized coordinates were predicted for several activity classes. However, most true positives were not separated from the bulk of ChEMBL screening compounds on the basis of potency predictions. Overall the ability of VS calculations to identify novel active compounds and separate them from false positives was only limited. Thus, although *de novo* structure generation from optimized coordinates is challenging, it would be difficult to replace the structure generation step in two-stage inverse QSAR with standard VS calculations. However, despite limited prediction accuracy, the VS calculations provided support for the chemical relevance of optimized coordinates because for each activity class, at least few true positives were among top-scoring screening compounds and proximal to optimized coordinates.

**Table 6.** Virtual screening details. Compound (CPD) statistics and VS results for distance-based compound rankings relative to optimized coordinates and potency-based rankings are reported.

TID	# ChEMBL compounds			AUC		True positive ratio (top 30 compounds)	
	Screening CPDs	CPDs in AD	True positive	Distance- based	Potency- based	Distance- based	Potency- based
11	1,413,665	1,004,761	497	0.76	0.51	0.30	0.13
15	1,412,983	1,020,546	1145	0.48	0.77	0.10	0.07
51	1,413,207	822,453	935	0.66	0.69	0.43	0.27
100	1,413,587	835,967	561	0.64	0.75	0.10	0.17
107	1,413,391	865,660	736	0.56	0.61	0.10	0.10
194	1,413,383	535,306	727	0.51	0.64	0.17	0.13
10193	1,412,986	1,172,027	1152	0.62	0.68	0.07	0.07
12209	1,413,301	942,956	838	0.55	0.73	0.00	0.00
12968	1,413,656	217,995	502	0.60	0.50	0.03	0.03

## Conclusions

The optimization of coordinates in feature space for high activity values predicted with a regression model is a central task in two-stage inverse QSAR. For this multi-constraint optimization problem, no generally applicable approach is currently available. The evaluation of differential evolution for coordinate optimization, as reported herein, was motivated by the successful application of this algorithm in areas of science other than chemistry. The study has provided proof-of-concept evidence that εDE is suitable for generating optimized coordinates in given feature spaces. For different compound classes, consistent predictions were obtained for εDE in combination with SVR, displaying robust convergence behavior and yielding optimized coordinates that not only met statistical and data set requirements, but were also chemically relevant, as indicated by compound mapping and distance- or potency-based VS calculations. However, due to limited prediction accuracy, distance-based VS relative to optimized coordinates would not be suitable to replace the *de novo* structure generation step in inverse QSAR, at least not on the basis of our reference calculations. Regardless, encouraging results were obtained for coordinate optimization. Taken together, the findings reported herein indicate that εDE optimization has the potential to further advance inverse QSAR analysis.

## Data availability

The data referenced by this article are under copyright with the following copyright statement: Copyright: © 2017 Miyao T et al.

ZENODO: Compound data sets for inverse QSAR and a descriptor list ^27^.

## Supplementary material

**Supplementary Figures S1-S3:** Prediction surfaces and PCA projections for different activity classes and prediction surfaces for the ChEMBL screening data set, respectively.
