# Supplementary material for: Exploring differential evolution for inverse QSAR analysis
Source: F1000Res. 2017 Sep 6;6:Chem Inf Sci-1285. Originally published 2017 Jul 31. [Version 2] doi: 10.12688/f1000research.12228.2 (PMC5580410; doi:10.12688/f1000research.12228.2)
Supplement: Supplementary file 1 [file f1000research-6-13622-s0000.tgz › 10d78515-6c8e-433c-81cb-5b8081cb6c84.docx]

**Supplementary material**

***Chemical Information Science Channel***

**Exploring differential evolution for inverse QSAR analysis**

Tomoyuki Miyao, Kimito Funatsu, and Jürgen Bajorath

**Supplementary Figures S1 – S3**

**Figure S1. Activity prediction.** For the all activity classes, predicted pK_i_ values are plotted against the scaled distance of the corresponding compounds to the optimized coordinates. Training data (cyan squares), test data (magenta squares), and optimized coordinates (green circle) are shown.

**
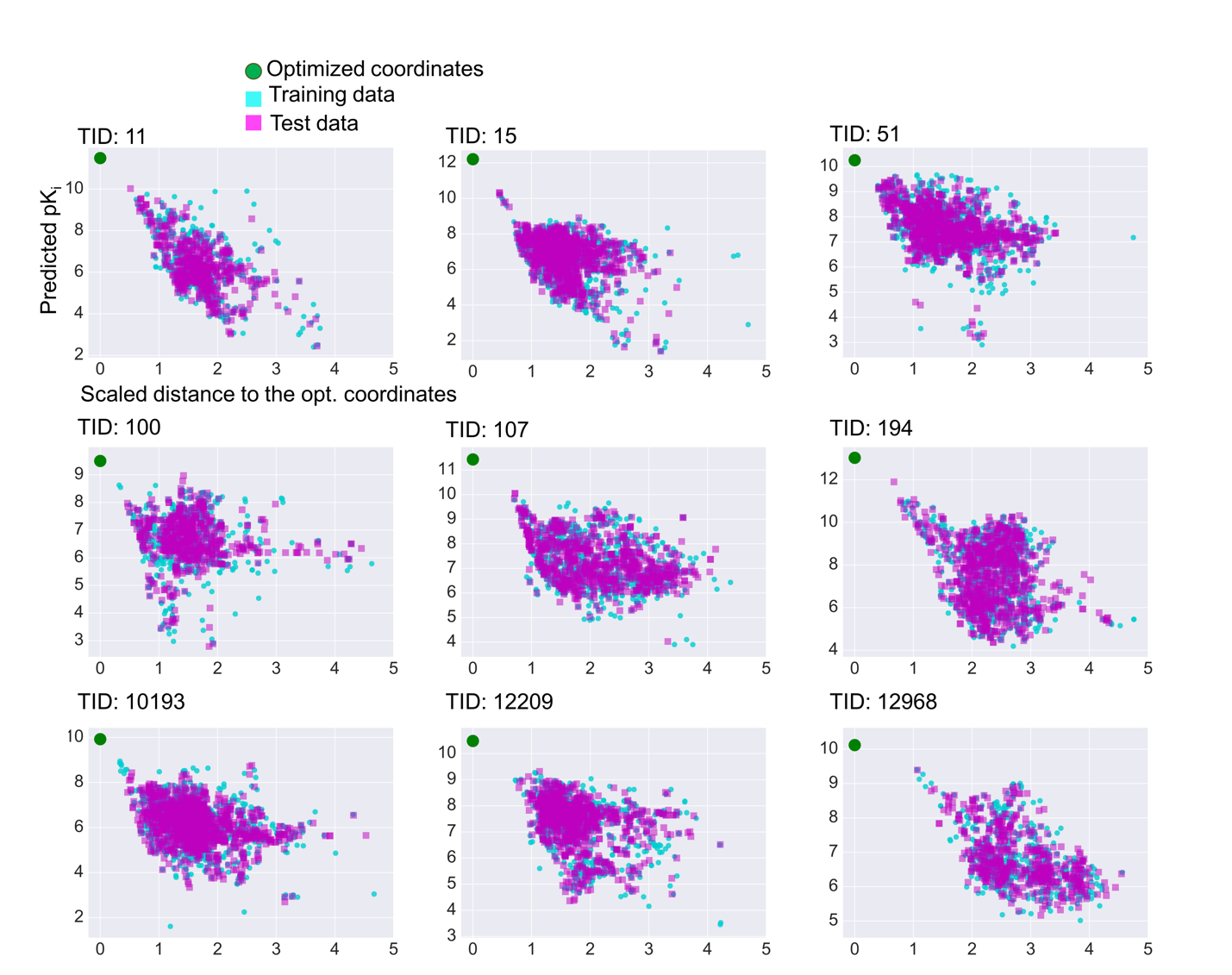
**

**Figure S2**. **Projection of optimized coordinates**. For all the activity classes, training data (cyan squares), test data (magenta squares), and optimized coordinates (green circle) are projected onto a principal component (PC) plot obtained from training data. For PC1 and PC2, contributions to data set variance are reported in %.

**
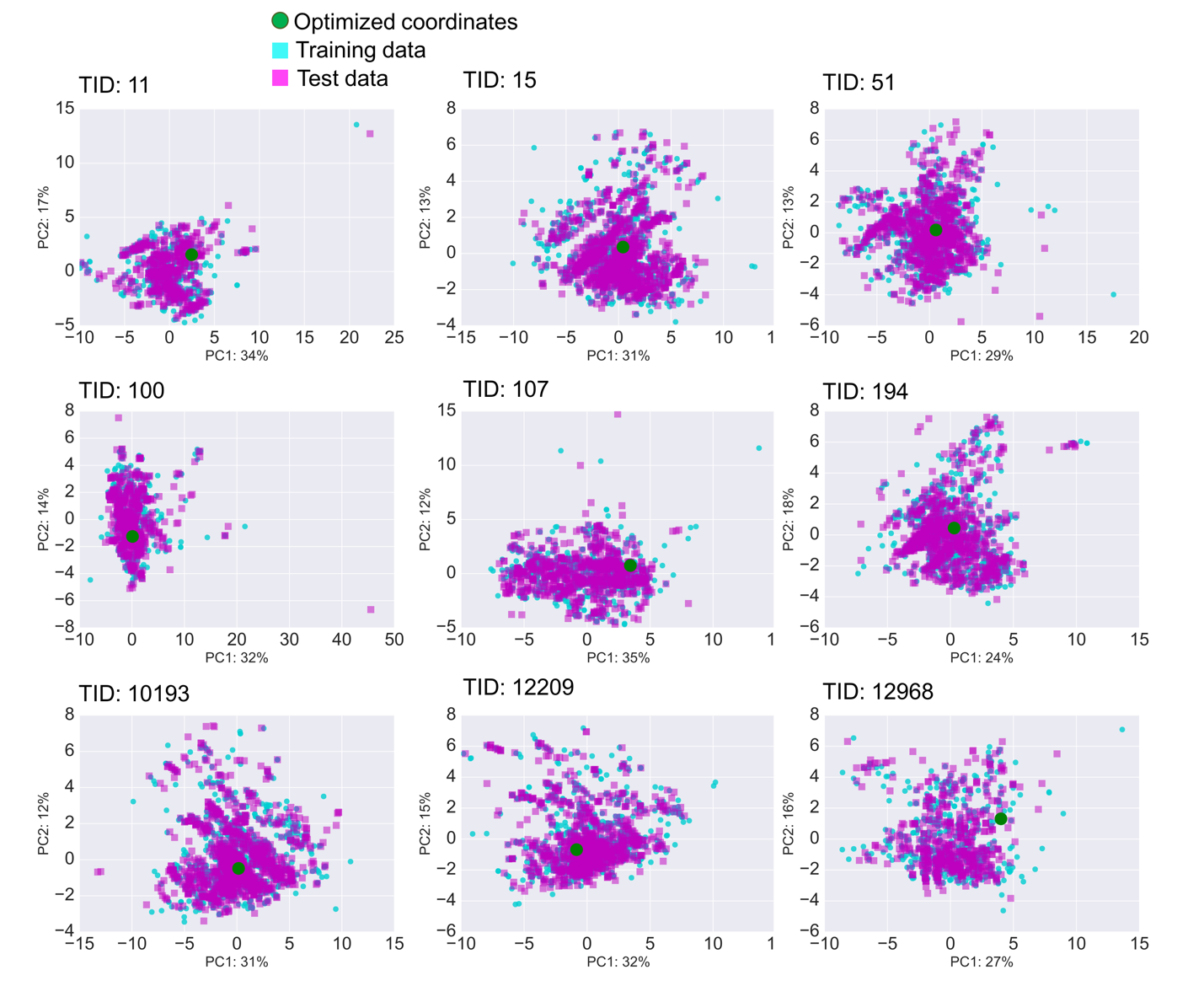
**

**Figure S3**. **Activity prediction for ChEMBL compounds.** For all activity classes, predicted pK_i_ values are plotted against the scaled distance of corresponding compounds to the optimized coordinates. ChEMBL compounds (cyan squares) and test compounds (true positives; magenta squares) falling inside the applicability domain are shown. Optimized coordinates are displayed as a green circle.

**
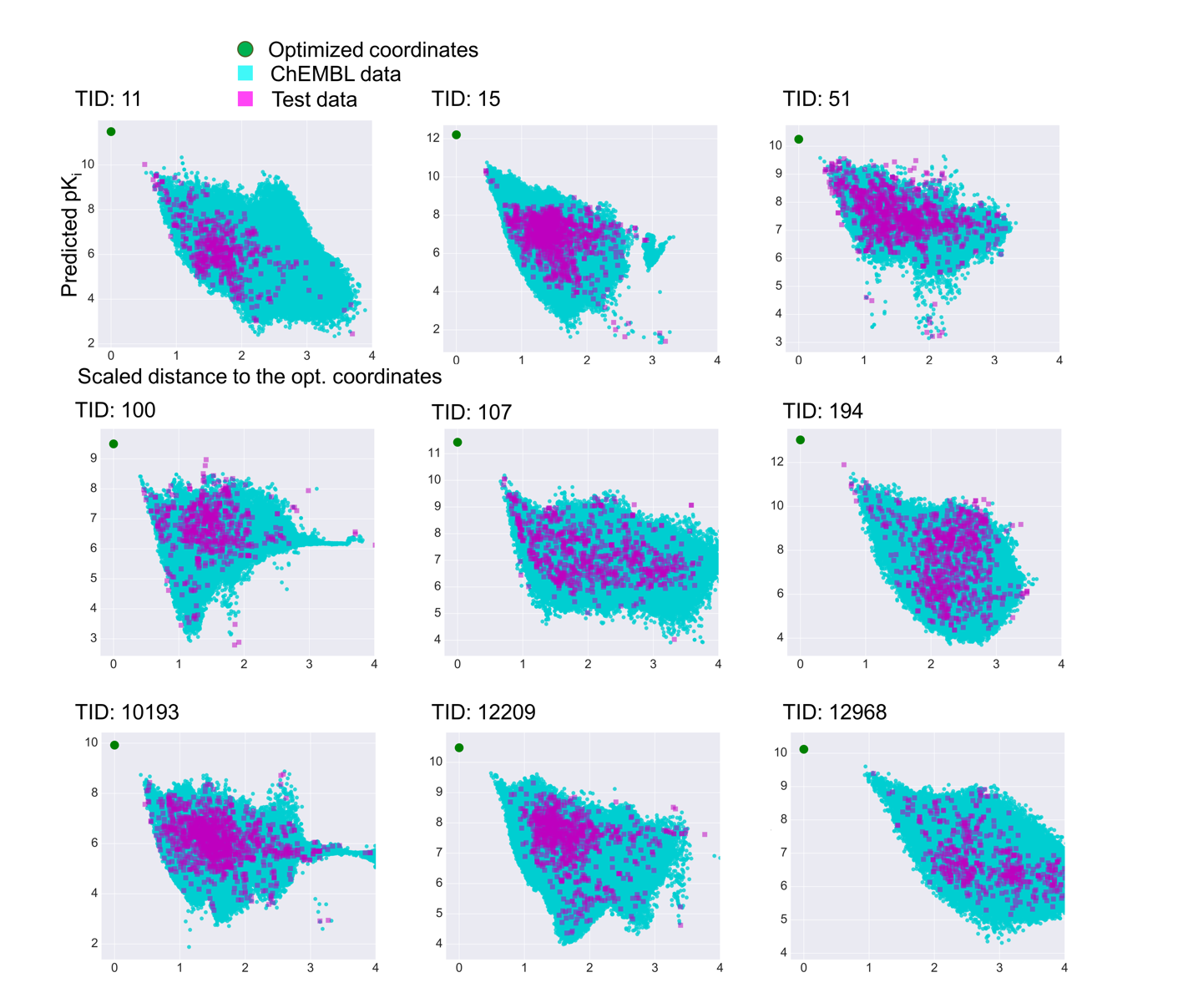
**
