# Supplementary material for: Exploring differential evolution for inverse QSAR analysis
Source: F1000Res. 2017 Sep 6;6:Chem Inf Sci-1285. Originally published 2017 Jul 31. [Version 2] doi: 10.12688/f1000research.12228.2 (PMC5580410; doi:10.12688/f1000research.12228.2)
Supplement: Peer review history

# Peer review: Referee response for version 1

**DOI:** 10.5256/f1000research.13238.r24662

**Published:** 2017-08-29

**Authors:** Patrick Walters

**Affiliations:** 1 Relay Therapeutics, Cambridge, MA, USA

This paper describes a new method for approaching one aspect of the inverse QSAR problem.  As the authors point out, the inverse QSAR problem can be divided into two steps.  

1. The generation of a set of coordinates, in some multidimensional space, that corresponds to one or more optimal compounds.  
2. The generation of molecular graphs that would produce chemical structures with descriptor values corresponding to these coordinates.  

This paper focuses on the first problem and does not address the second. 

 The paper is well written and the topic will be of interest to computational chemists working in both industry and academia.  The methodology is described well and an individual with some QSAR expertise should be able to reproduce this work.  However, in the interest of making it easier to reproduce the work described here, and making the method more widely available, it would be useful for the authors to make a reference implementation available.  On a similar note, the authors mention that the ChEMBL datasets are available from the original source.  As a service to those readers who would like to reproduce the work, it would be useful if the authors provided the specific datasets used in this paper as a download.  As part of this download, the authors could also include a list of specific descriptors used and annotate which compounds were rejected due to PAINS filters.  

 The definition of the applicability domain used in this paper could be expanded.  It would be useful for the authors to provide a specific worked example demonstrating how the applicability domain is defined for one of the ChEMBL examples described in the paper.  This example could be expanded to demonstrate how a set of optimal coordinates is defined. 

 One other beneficial addition to this paper would be a comparison with established methods.  The authors provide the results of virtual screening based on their method but do not provide a comparison with commonly used techniques.  One way to do this would be to provide a simple comparison with an SVM model for activity calculated based on the same descriptions.  A comparison with activity calculated based on nearest neighbors in a simple principal component space could also be provided.  

 A few specific comments:

 It is unclear what the RMSE value is in Table 2.  This is on an arbitrary dataset, how should RMSE be interpreted? 

 There appear to be a number of errors in the chemical structures in Figure 7.

I have read this submission. I believe that I have an appropriate level of expertise to confirm that it is of an acceptable scientific standard.

# Peer review: Referee response for version 1

**DOI:** 10.5256/f1000research.13238.r24700

**Published:** 2017-08-25

**Authors:** Brian Goldman

**Affiliations:** 1 Modeling & Informatics, Vertex Pharmaceuticals, Boston, MA, USA

This is an extremely well written submission by Bajorath *et. al* detailing an important aspect of the inverse QSAR problem, namely the generation of a feature vector optimizing the output of a QSAR equation.  An interesting and novel component of the work is that constrained optimization is utilized ensuring generated solutions lie within the domain of applicability of the QSAR model.  This paper is primarily of theoretical interest in that due to inherent limitations with inverse-QSAR the technique is rarely adopted as a means of drug discovery.  The primary reason for the lack of adoption revolving around the de-novo design of compounds matching optimized descriptor values.

 The introduction to the paper covers the relevant literature but could be made stronger by discussing the recent work of Gómez-Bombarelli *et. al.* ^1^ **who use generative models to approach the inverse-QSAR problem.  Their work concerns building and optimizing QSAR equations in the latent space of an autoencoder and subsequently decoding optimized points into molecular structures.  Although the work by Gómez-Bombarelli is preliminary, it addresses both QSAR optimization and structure generation and would most likely be interesting to readers of the current paper.

 An aspect of the current technique that could be discussed in the paper is the generation of a family of solutions.  Currently, the presented technique produces one optimized feature vector.  It would strengthen the paper if the authors discussed how a family of diverse feature vectors (structures) could be evolved using the presented methodology. 

 Overall, the paper in its current form is more than acceptable. The methodology is clearly delineated and sufficiently supported by the included results.

 Minor points:

- Figure 3: it would be informative to highlight the optimal point with a particular glyph (perhaps a red star?)
- Figure 7: Chemical structures look suspect and should be checked

I have read this submission. I believe that I have an appropriate level of expertise to confirm that it is of an acceptable scientific standard.

## References

[1] Gómez-BombarelliRDuvenaudDHernández-LobatoJMAguilera-IparraguirreJHirzelTDAdamsRPAspuru-GuzikA: Automatic chemical design using a data-driven continuous representation of molecules. *arXiv*.2017; Reference source 10.1021/acscentsci.7b00572PMC583300729532027

# Peer review: Referee response for version 1

**DOI:** 10.5256/f1000research.13238.r24665

**Published:** 2017-08-09

**Authors:** Hans Matter

**Affiliations:** 1 R&D IDD / Structure, Design & Informatics, Sanofi-Aventis Deutschland GmbH, Frankfurt, Germany

This interesting contribution by Bajorath *et al. *addresses an important part of the inverse QSAR problem towards automated generation of structures with high activity from QSAR models. Inverse-QSAR, while intellectually appealing, does not find significant applications in modelling in the pharmaceutical industry. Main limitations are linked to de-novo structure generation due to issues with synthetic accessibility. Another hurdle is the challenge to identify the optimal descriptor setting for a model. The authors focus on this latter point, namely a novel and accurate approach for coordinate optimization. They demonstrate how to generate optimal descriptor coordinates under certain constraints like model applicability domain and meaningful descriptor values. A convincing validation study using virtual screening in ChEMBL 22 is also presented.

 The report title and abstract cover the content well. The chemoinformatics approach is well conducted and clearly described. The results are presented in a clear and interesting way and capture the interest of F1000 readers. The authors might also want to mention, whether software tools and subroutines from their study are available. Therefore this contribution is an essential view on interpretation of QSAR models and should be indexed in its present form.

 Some points could be addressed to highlight further aspects of their work. 

- How does such an optimization approach handle typical types of variables from real-life models, e.g. two-level variables, variables with small or no SD?
- Often model analysis should not result in a single solution, but multiple related structures. Could the optimization approach find multiple descriptor regions to offer options for monitoring secondary properties (e.g. solubility)?
- It might be illustrative for one ChEMBL target to systematically generate analogs for potent leads close to the descriptor optimum by applying simple MedChem transformations and check, whether some analogs come closer to the optimum in descriptor space. Chemical changes are minimal here and one could access their impact to the descriptor optimum.
- The moderate VS success using QSAR models might suggest a non-optimal approach to define the applicability domain. Some details on the AD definition and the descriptor space might be useful. Do the authors expect that a more strict AD definition might produce reliable results? What does this mean for de-novo structure generation as second step?
- Is it possible to apply such a concept for multi-parameter optimization, e.g. multiple QSAR models combined for predicting compound profiles / selectivity / druglikeness?
- Minor point: Drawings of chemical structures in figure 7 need to be checked.

I have read this submission. I believe that I have an appropriate level of expertise to confirm that it is of an acceptable scientific standard.
